# Infusion of Plasma from Exercised Mice Ameliorates Cognitive Dysfunction by Increasing Hippocampal Neuroplasticity and Mitochondrial Functions in 3xTg-AD Mice

**DOI:** 10.3390/ijms21093291

**Published:** 2020-05-06

**Authors:** Tae-Woon Kim, Sang-Seo Park, Joon-Young Park, Hye-Sang Park

**Affiliations:** 1 Exercise Rehabilitation Research Institute, Department of Exercise & Health Science, Sangmyung University, Seoul 03016, Korea; twkim0806@naver.com; 2 Department of Physiology, College of Medicine, KyungHee University, Seoul 02447, Korea; sps07@naver.com; 3 Department of Kinesiology, College of Public Health and Cardiovascular Research Center, Lewis Katz school of Medicine, Temple University, Philadelphia, PA 19122, USA; parkjy@temple.edu

**Keywords:** Alzheimer’s disease, cognitive function, hippocampus, neuroplasticity, mitochondria, young plasma, exercise

## Abstract

Alzheimer’s disease is the most common neurodegenerative brain disease causing dementia. It is characterized by slow onset and gradual worsening of memory and other cognitive functions. Recently, parabiosis and infusion of plasma from young mice have been proposed to have positive effects in aging and Alzheimer’s disease. Therefore, this study examined whether infusion of plasma from exercised mice improved cognitive functions related to the hippocampus in a 3xTg-Alzheimer’s disease (AD) model. We collected plasma from young mice that had exercised for 3 months and injected 100 µL of plasma into the tail vein of 12-month-old 3xTg-AD mice 10 times at 3-day intervals. We then analyzed spatial learning and memory, long-term memory, hippocampal GSK3β/tau proteins, synaptic proteins, mitochondrial function, apoptosis, and neurogenesis. In the hippocampus of 3xTg-AD mice, infusion of plasma from exercised mice improved neuroplasticity and mitochondrial function and suppressed apoptosis, ultimately improving cognitive function. However, there was no improvement in tau hyperphosphorylation. This study showed that plasma from exercised mice could have a protective effect on cognitive dysfunction and neural circuits associated with AD via a tau-independent mechanism involving elevated brain-derived neurotrophic factor due to exercise.

## 1. Introduction

Alzheimer’s disease (AD) is the most common cause of dementia, characterized by slow onset and progressive decline of memory and cognitive functions. AD is associated with various cellular changes in the brain, including synaptic injury, alterations in mitochondrial structure and function, abnormal inflammatory response, extracellular accumulation of amyloid beta (Aβ), and intracellular neurofibrillary tangles [1,2,3]. AD is caused by atrophy, senile plaques, and hyperphosphorylated tau protein aggregates in the hippocampus, one of the neuroanatomical areas responsible for memory and learning [4,5]. Cognitive dysfunction, including that involving memory and learning, is associated with decreased neurogenesis in the hippocampus, which could result from decreased expression of immature neuron factors, such as DCX (doublecortin), that signal the birth of new neurons [6].

Tau overexpression and hyperphosphorylation have been found to impair axonal movement of cell organelles, including mitochondria [7,8,9]. There has been much evidence demonstrating mitochondrial dysfunction accompanied by aging and aging-related neurodegenerative disease [10]. In particular, mitochondrial dysfunction has been observed in fibroblasts and hematopoietic cells from the brains of AD transgenic mouse models and human patients with AD [11,12,13,14]. It has been suggested that mitochondrial dysfunction could develop when the course of AD worsens or in all stages of the disease, and that this process may occur not only in the brain, but systemically [13].

In recent research on aging-related treatments, injection of young mouse blood into aged mice has shown positive effects, which may be applied as a new experimental approach for the treatment of aging-related diseases. According to previous studies, parabiosis between 18-month-old and 5-week-old mice resulted in increased expression of genes related to brain activity than in mice that did not receive blood transfusions, especially immediate-early genes in hippocampal cells [15]. This indicated that parabiosis increased brain activity and improved memory. With increasing age, the synapses, which form the network for communication between neurons, begin to regress, leading to degeneration of neurons, atrophy of the brain, and a sudden increase in neurodegenerative disease. Blood links the diverse tissues of the body. Blood is not only a transport medium for oxygen and cells, but also helps to fight infectious disease and conveys information in the form of hormones and other molecules. In other words, blood plays an important role in conveying information between cells and tissues, including the brain.

Meanwhile, exercise not only has a positive effect in patients with neurodegenerative diseases such as AD and Parkinson’s disease, but also has positive effects on plasma. Exercise-induced neuroplasticity, characterized by inhibited apoptosis and increased neurogenesis and brain-derived neurotrophic factor (BDNF), facilitates recovery from brain damage after traumatic brain injury, ischemia, and stroke [16,17,18,19]. The plasma concentration of BDNF increases after aerobic exercise [20], and exercise training has also been reported to increase resting plasma BDNF [21]. As AD worsens, it eventually leads to loss of motor function. Although exercise has various positive effects on the brain, the drawback is that these effects are only possible when the individual is capable of physical activity. Thus, in this study, we aimed to examine whether transfusion of plasma from exercised mice could have similar effects to exercise on cognitive function, hippocampal neuroplasticity, and mitochondrial function in AD.

## 2. Results

### 2.1. Effect of Exercise on Plasma BDNF in Donation Mice

**Figure 1:**
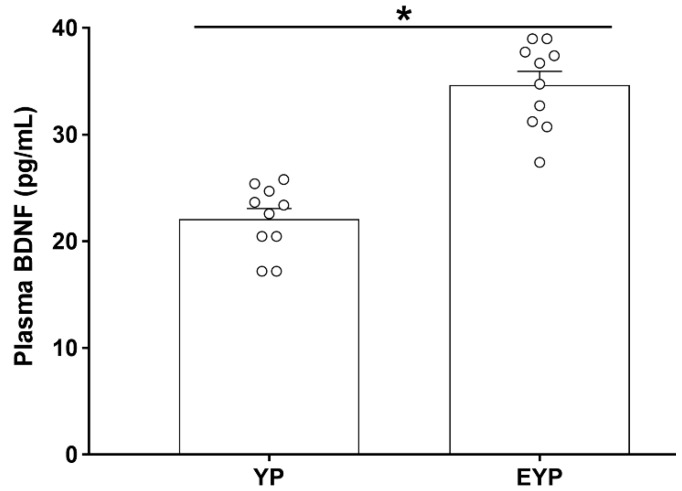
Effect of exercise on plasma brain-derived neurotrophic factor (BDNF) in donation plasma mice. YP: young plasma, EYP: exercised young plasma. Data are expressed as the mean ± standard error of the mean (SEM). * *p* < 0.05.

Enzyme-linked immunosorbent assay (ELISA) was performed to investigate changes in plasma BDNF concentration. The group treated with exercised young plasma (EYP) showed significantly increased plasma BDNF concentrations compared with the group treated with young plasma (YP) (*p* < 0.001) (Figure 1).

### 2.2. Effect of Plasma from Young Exercised Mice on Cognitive Functions in 3xTg-AD Mice

**Figure 2:**
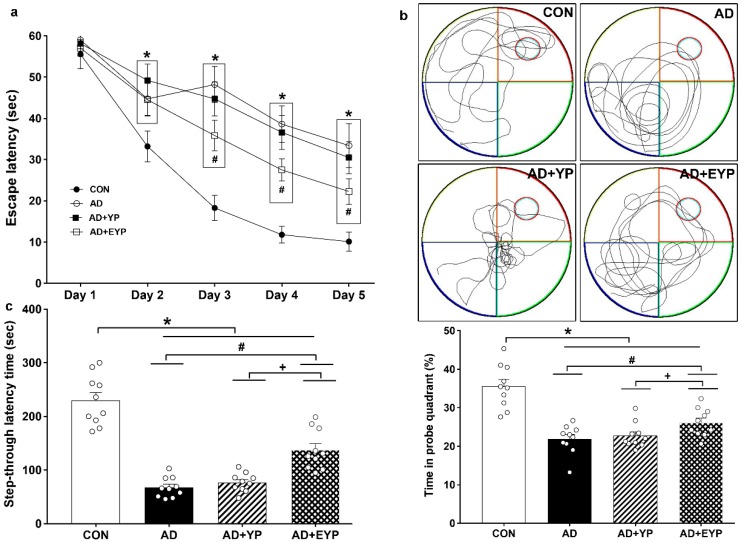
Effect of plasma from young exercised mice on cognitive functions in 3xTg-AD mice. The Morris water maze task for spatial learning (**a**) and memory (**b**) and step through task for long-term memory (**c**). CON: wild-type, AD: 3xTg-AD, AD + YP: 3xTg-AD and young plasma injection, AD + EYP: 3xTg-AD and exercised young plasma injection group. Data are expressed as the mean ± standard error of the mean (SEM). * *p <* 0.05 compared with the CON group. # *p <* 0.05 compared with the AD group. + *p* < 0.05 between AD + YP and AD + EYP groups.

To assess spatial learning and long-term memory, the Morris water maze and step-through avoidance tasks were performed. Spatial learning was assessed by measuring the time on the platform. In spatial learning ability, the AD group took longer to find the platform from day 2 compared to the control (CON) group (*p* < 0.001). The group treated with EYP took less time to find the platform than the AD group starting from day 3 (Day 3: *p* = 0.039, Day 4: *p* = 0.017, Day 5: *p* < 0.001) (Figure 2a). The AD group showed reduced spatial memory (*p* < 0.001) and long-term memory (*p* < 0.001) compared to the CON group. The group treated with YP infusion did not demonstrate statistically significant differences in these tests; however, infusion of plasma from young exercised mice improved spatial memory and long-term memory compared with the AD group (*p* = 0.008 and *p* = 0.030, respectively). Treatment comparisons revealed significant differences in spatial and long-term memory between AD + YP and AD + EYP combined groups (*p* < 0.001, *p =* 0.028, respectively) (Figure 2b,c). Infusion of plasma from exercised young mice conferred positive effects in improving spatial memory, learning ability, and long-term memory.

### 2.3. Effect of Plasma from Young Exercised Mice on GSK3β and Tau Protein Expression in Hippocampus

**Figure 3:**
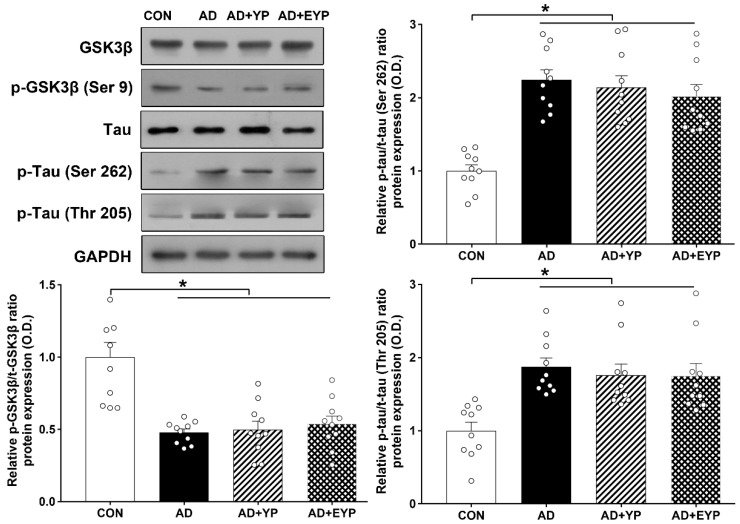
Effect of plasma from young exercised mice on GSK3β and tau protein expression in the hippocampus. CON: wild-type, AD: 3xTg-AD, AD + YP: 3xTg-AD and young plasma injection, AD + EYP: 3xTg-AD and exercised young plasma injection group. Data are expressed as the mean ± standard error of the mean (SEM). * *p <* 0.05 compared with the CON group.

Western blot analysis was performed to investigate changes in GSK3β/tau protein in the hippocampus. For group comparisons, results were normalized relative to the CON group. AD, AD + YP, and AD + EYP groups showed significantly decreased p-GSK3β/GSK3β ratios (*p* < 0.001) and significantly increased p-tau (Ser262, Thr205)/tau ratios (*p* < 0.001) compared with the CON group. Differences between groups not including the CON group were not statistically significant (Figure 3).

### 2.4. Effect of Plasma from Young Exercised Mice on Mitochondrial Ca^2+^ Retention and H_2_O_2_ Emission in the Hippocampus

**Figure 4:**
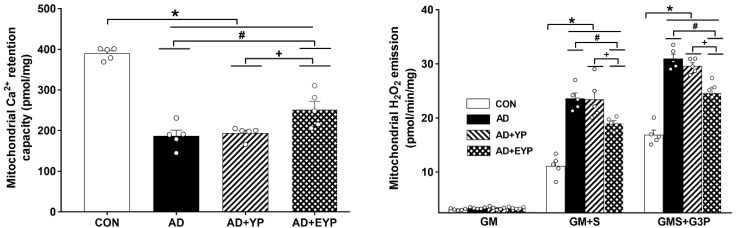
Effect of plasma from young exercised mice on mitochondrial Ca^2+^ retention and H_2_O_2_ emission in hippocampus. CON: wild-type, AD: 3xTg-AD, AD + YP: 3xTg-AD and young plasma injection, AD + EYP: 3xTg-AD and exercised young plasma injection group. Data are expressed as the mean ± standard error of the mean (SEM). * *p <* 0.05 compared to the CON group. # *p <* 0.05 compared to the AD group. + *p* < 0.05 between the AD + YP and AD + EYP groups.

AD, AD + YP, and AD + EYP groups showed significantly reduced mitochondrial Ca^2+^ retention capacity in the hippocampus compared to the CON group (*p* < 0.001). Infusion of plasma from young exercised mice resulted in a significant increase in mitochondrial Ca^2+^ retention capacity in the hippocampus compared to the AD group (*p* = 0.034) and the AD + YP group (*p* = 0.021). The AD group and the AD + YP group did not show statistically significant differences (Figure 4 left). The mitochondrial H_2_O_2_ emission rate was calculated for complex I substrates (glutamate + malate, GM), complex 2 substrates (succinate, GM + S), and lipid substrate (glycerol-3 phosphate, GMS + G3P). Mitochondrial H_2_O_2_ emission rate in hippocampal tissue was significantly increased with complex 2 substrates (succinate, GM + S) and lipid substrates (glycerol-3 phosphate, GMS + G3P) in the AD, AD + YP, and AD + EYP groups compared with that in the CON group (*p* < 0.001). Infusion of plasma from young exercised mice resulted in significant decreases in all substrates except for complex 1 substrate (GM + S: *p* = 0.032, GMS + G3P: *p* = 0.013). There were significant differences in H_2_O_2_ emission rate between AD + YP and AD + EYP combination groups (*p* < 0.001, respectively). The AD group and the AD + YP group did not show statistically significant differences (Figure 4 right). Thus, infusion of plasma from young exercised mice helped to maintain mitochondrial calcium homeostasis and reduced reactive oxygen species (ROS), resulting in improved mitochondrial function in the hippocampus.

### 2.5. Effect of Plasma from Young Exercised Mice on Apoptosis in the Hippocampus

**Figure 5:**
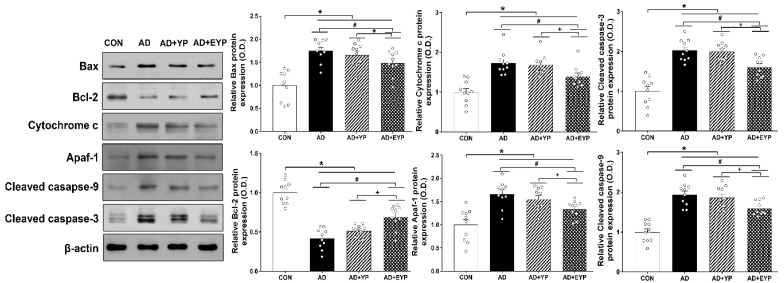
Effect of plasma from young exercised mice on apoptosis in the hippocampus. CON: wild-type, AD: 3xTg-AD, AD + YP: 3xTg-AD and young plasma injection, AD + EYP: 3xTg-AD and exercised young plasma injection group. Data are expressed as the mean ± standard error of the mean (SEM). * *p <* 0.05 compared to the CON group. # *p <* 0.05 compared to the AD group. + *p* < 0.05 between AD + YP and AD + EYP groups.

To investigate changes in apoptotic proteins in the hippocampus, we analyzed the expression levels of Bax, Bcl-2, cytochrome c, apaf-1, and cleaved caspase-3 and -9. To investigate cell death, we analyzed the number of TUNEL-positive cells. To compare cell death between groups, the CON group results were set as 1, and the other groups’ results were normalized relative to the CON group. AD, AD + YP, and AD + EYP groups showed significant increased expressions of Bax, cytochrome c, and cleaved caspase-3 and -9, and significant decreased expression of Bcl-2 compared to the CON group (*p* < 0.001). The AD + EYP combination group demonstrated significantly decreased expression of Bax (*p* = 0.038), cytochrome c (*p* < 0.001), apaf-1 (*p* = 0.033), and cleaved caspase-3 and -9 (*p* = 0.041, *p* = 0.023, respectively), and significantly increased expression of Bcl-2 (*p* = 0.039) compared with the AD group. There were significant differences in apoptosis between AD + YP and AD + EYP groups (Bax: *p* = 0.034, Bcl-2: *p* = 0.015, cytochrome c: *p* = 0.028, apaf-1: *p* = 0.039), cleaved caspase-3: *p* = 0.029 and -9: *p* = 0.020). The AD group and the AD + YP group did not show statistically significant differences (Figure 5). Thus, plasma from young exercised mice inhibited apoptosis and cell death in the hippocampus of AD mice.

### 2.6. Effect of Plasma from Young Exercised Mice on Cell Death in the Hippocampal Dentate Gyrus

**Figure 6:**
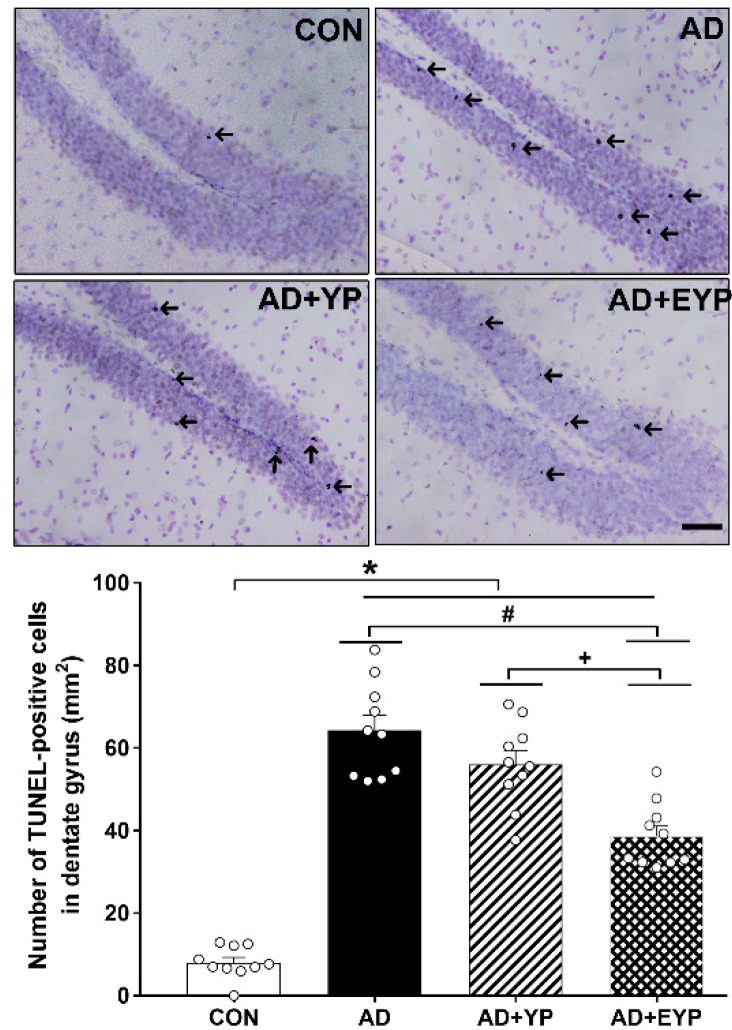
Effect of plasma from young exercised mice on cell death in the hippocampal dentate gyrus (DG). Photomicrographs and data of TUNEL-positive cells (arrow). The scale bar represents 50 μm. CON: wild-type, AD: 3xTg-AD, AD + YP: 3xTg-AD and young plasma injection, AD + EYP: 3xTg-AD and exercised young plasma injection group. Data are expressed as the mean ± standard error of the mean (SEM). * *p <* 0.05 compared to the CON group. # *p <* 0.05 compared to the AD group. + *p* < 0.05 between AD + YP and AD + EYP groups.

To investigate cell death in the hippocampal dentate gyrus (DG) we utilized a TUNEL assay. AD, AD + YP, and AD + EYP groups showed a significantly increased numbers of hippocampal TUNEL-positive cells compared to the CON group (*p* < 0.001). The AD + EYP group demonstrated a significantly reduced number of TUNEL-positive cells compared to the AD group (*p* = 0.012) and the AD + YP group (*p* = 0.011), whereas there was no significant difference between the AD and AD + YP groups (Figure 6). Thus, plasma from young exercised mice inhibited cell death in the hippocampus of AD mice.

### 2.7. Effect of Plasma from Young Exercised Mice on the Expression of BDNF, PSD 95, and Synaptophysin in the Hippocampus

**Figure 7:**
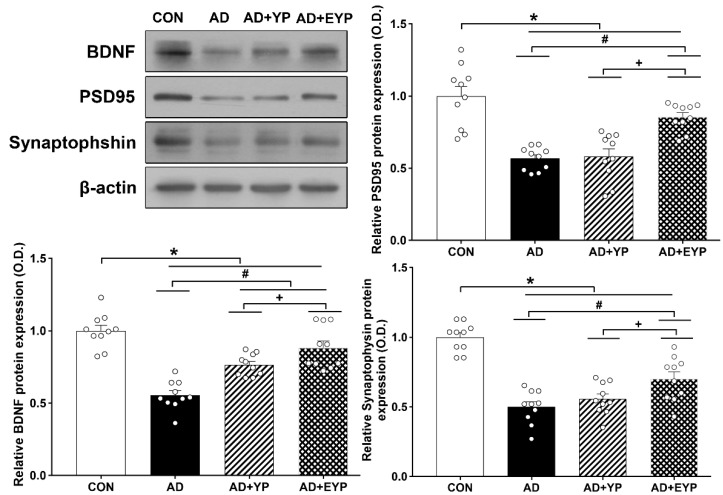
Effect of plasma from young exercised mice on the expressions of BDNF, PSD 95, and synaptophysin in the hippocampus. CON: wild-type, AD: 3xTg-AD, AD + YP: 3xTg-AD and young plasma injection, AD + EYP: 3xTg-AD and exercised young plasma injection group. Data are expressed as the mean ± standard error of the mean (SEM). * *p <* 0.05 compared to the CON group. # *p <* 0.05 compared to the AD group. + *p <* 0.05 compared AD + YP and AD + EYP.

To investigate synaptic proteins in the hippocampus, we analyzed the protein expression levels of BDNF, PSD 95, and synaptophysin. For comparisons between groups, results were normalized to the CON group. AD, AD + YP, and AD + EYP groups showed decreased expressions of BDNF, PSD 95, and synaptophysin proteins compared to the CON group (all *p* < 0.001). The AD + EYP group demonstrated increased protein levels of BDNF (*p* < 0.001), PSD 95 (*p* < 0.001), and synaptophysin (*p* = 0.025) compared to the AD group. There were significant differences in expression of synaptic proteins between the AD + YP group and the AD + EYP group (BDNF: *p* = 0.040, PSD95: *p* = 0.018, synaptophysin: *p* = 0.024). The AD + YP group showed a significant increase in BDNF protein expression (*p* = 0.030), whereas PSD95 and synaptophysin expression were not statistically significant compared to the AD group (Figure 7). Thus, infusion of plasma from young control and exercised mice increased the levels of synaptic proteins in the hippocampus of AD mice.

### 2.8. Effect of Plasma from Young Exercised Mice on Cell Proliferation and Neurogenesis in the Hippocampus

**Figure 8:**
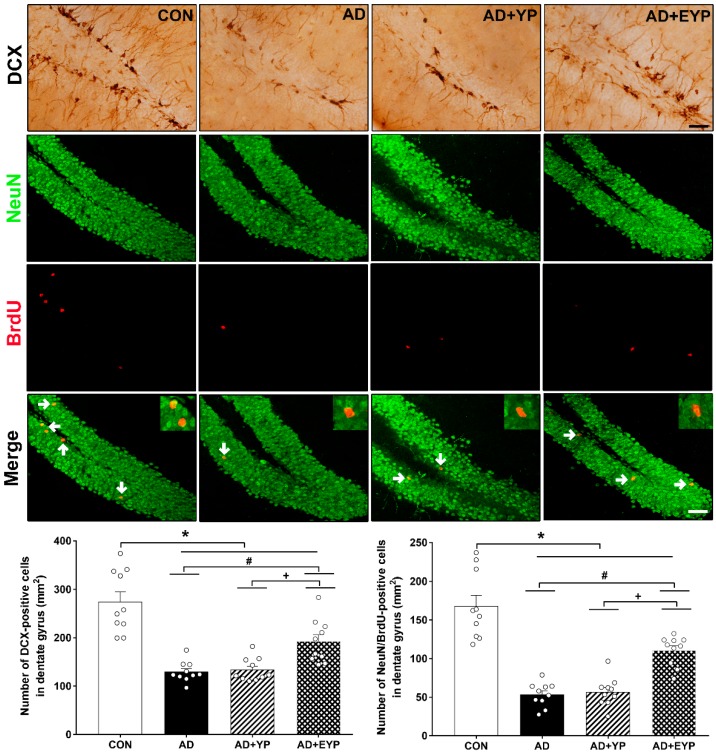
Effect of plasma from young exercised mice on cell proliferation and neurogenesis in the hippocampus. CON: wild-type, AD: 3xTg-AD, AD + YP: 3xTg-AD and Young plasma injection, AD + EYP: 3xTg-AD and exercised young plasma injection group. Photomicrographs and data of DCX- and NeuN/BrdU-positive cells (white arrows). The scale bar represents 50 μm. Data are expressed as the mean ± standard error of the mean (SEM). *, *p <* 0.05 compared to the CON group. #, *p <* 0.05 compared to the AD group. *+, p <* 0.05 between AD + YP and AD + EYP groups.

To investigate cell proliferation and neurogenesis in the hippocampus, we analyzed DCX expression-positive cells (marker of cell proliferation) and NeuN/brdU-positive cells. AD, AD + YP, and AD + EYP groups showed reduced hippocampal levels of DCX-positive cells and NeuN/brdU-positive cells (*p* < 0.001, respectively) compared to the CON group. The AD + EYP group demonstrated increased levels of DCX-positive cells (*p* = 0.014) and NeuN/brdU-positive cells (*p* = 0.041) in the hippocampus compared to the AD group. There were significant differences in the numbers of DCX and NeuN/brdU-positive cells between the AD + YP and the AD + EYP group (*p* = 0.010, *p* = 0.009, respectively). The AD + YP group did not show statistically significant differences compared to the AD group (Figure 8). Thus, infusion of plasma from exercised young mice increased neurogenesis in the hippocampus.

## 3. Discussion

Increasing evidence suggests that AD pathogenesis is not restricted to the neuronal compartment. Glial cells, particularly reactive astrocytes and activated microglia, appear to play critical and interactive roles in neurodegeneration [22,23]. Accumulating evidence of their role in brain energy metabolism and reduced oxygen supply to the brain clearly point to their critical involvement in the prevention, initiation, and progression of neurodegenerative diseases, including AD [10,24]. However, AD is an age-related neurodegenerative disease caused by accumulation of Aβ, neurofibrillary tangles, gradual nerve injury, and cognitive deficits. In 3xTg mice, we observed pathological elements similar to those of AD, including tau hyperphosphorylation and impaired spatial memory, which are consistent with previous studies [25]. These symptoms have been reported to gradually worsen over time [26]. Among the pathological elements of AD, overexpression and phosphorylation of tau is correlated with mitochondrial dysfunction including ROS production, reduced ATP, fragmenting of mitochondrial membrane potential, and ultimately, neuronal damage [27,28]. Specifically, elevated levels of ROS, including H_2_O_2_, resulting from impaired regulation of mitochondrial Ca^2+^ homeostasis have been shown to increase the sensitivity of mitochondrial PTP opening [29], which in turn promotes apoptosis [30]. In the present study, the AD group showed tau hyperphosphorylation, reduced levels of synaptic plasticity markers such as BDNF, PSD95, and synaptophysin, reduced cell proliferation and neurogenesis, reduced mitochondrial Ca^2+^ retention, and elevated H_2_O_2_. There were increased levels of Bax, apaf-1, cytochrome c, cleaved caspase-3 and -9, decreased Bcl-2 levels, and increased cell death in the AD group. In the hippocampus, tau hyperphosphorylation impairs cognitive function by not only inhibiting cell proliferation and neurogenesis, but also by increasing the number of cells undergoing apoptosis via mitochondrial dysfunction.

Various studies have demonstrated that exercise has positive effects on brain health, and exercise is especially known to play a beneficial role in AD treatment. However, the obvious but essential precondition for exercise is that the individual must physically able. AD is known to start as mild cognitive impairment and eventually progress to loss of motor function, eliminating the possibility of exercise. However, recent research on aging has suggested that parabiosis could have positive effects on cognitive function in the elderly. When plasma from young mice was infused into aged mice, behavioral tests, including those for contextual fear conditioning, spatial learning, and memory showed improvements in aging-related cognitive dysfunction [15]. Conversely, when plasma from elderly mice was infused into young mice, neurogenesis, learning, and memory decreased [31]. Yuan et al. [32] reported that treatment with plasma from young mice reduced acute brain injury induced by aging-related hemorrhagic stroke. In one recent study, parabiosis and intravenous infusion of plasma from young mice resulted in near-complete recovery of synaptic and neural protein levels in an animal model of AD [33]. These results suggest that factors carried in the blood might partially contribute to synaptic plasticity, neurogenesis, and cognitive function. In the present study, the young plasma group showed partial, trend-level improvements in hippocampal GSKβ/Tau expression, neuroplasticity, and mitochondrial function, but these were not statistically significant. Conversely, the group infused with plasma from mice that had exercised for 12 weeks showed overall improvements accompanied by positive effects on cognitive function. The effects of plasma from exercised mice may be due to elevated levels of BDNF in the blood resulting from exercise. Among the young plasma donor mice in this study, those that had exercised showed higher levels of plasma BDNF than those that had not exercised. Previous animal and clinical studies have also shown that blood BDNF levels increase after exercise [34,35]. Qin et al. [36] reported that reduced peripheral blood BDNF is important clinical evidence of AD or mild cognitive impairment, supporting a relationship between decreasing BDNF level and AD progression. Specifically, plasma BDNF level can reflect hippocampal BDNF. Therefore, plasma BDNF level is an important and stable blood biomarker of AD [37,38].

From a neuroprotective stance, BDNF secreted at the synaptic cleft modulates neuroplasticity to confer protection from neural cell death caused by Aβ aggregates and tau protein, which are involved in the pathology of AD [39]. In a study by Jiao et al. [40], delivery of the BDNF gene was proposed to be a good treatment method for tau-related neural degeneration in AD and other tauopathy-related neurodegenerative diseases. BDNF is involved in the control of neurogenesis; normal BDNF-TrkB signaling is essential for the long-term survival of new neurons in the hippocampal dentate gyrus [41] and can also inhibit caspase-3 activity as a result of apoptotic stimuli [42]. Mitochondrial function in the brain is increased by BDNF in a dose-dependent manner, and BDNF counteracts damage caused by mitochondrial Ca^2+^ overload [43]. However, in the present study, plasma from young control and exercised mice failed to improve tau protein levels. In previous studies, heterochronic parabiosis and infusion of plasma from young mice improved synaptic and neuronal proteins as well as cognitive function in mutant mice for amyloid precursor protein without a decrease in Aβ burden [33]. Meanwhile, BDNF supplementation relieved behavior deficits, protected against neuronal loss, and alleviated synaptic degeneration and neuronal abnormality in P301L mutant tau transgene mice, but had no effect on tau hyperphosphorylation [40]. Consistent with previous studies on plasma infusion and BDNF, the present study found that infusion of plasma from exercised mice in an AD model did not reduce tau hyperphosphorylation but did enhance cognitive function via improvements in hippocampal synaptic proteins, neuroplasticity, including apoptosis, and mitochondrial function.

## 4. Material and Methods

### 4.1. Animals

All animal experiments were conducted according to the National Institutes of Health and National Institutes of Health and Korean Academy of Medical Sciences guidelines. The study protocol was approved by the Kyung Hee University Animal Care and Use Committee (Approval No. KHUASP [SE] -17-103, 14 August 2017). The mice were kept under constant temperature (25 ± 1 °C) and light (7 AM to 7 PM) conditions, and food and water were provided arbitrarily. The 12-month-old male mice were randomly divided into the following groups: wild type (CON), 3xTG-AD (AD), 3xTg-AD, young plasma injection (AD + YP), and 3xTg-AD and exercised young plasma injection (AD + EYP) groups (*n* = 10 in each group). The plasma used for the donation was obtained from 4-month-old C57BL/6 mice from the same mouse line that had undergone a 12-week exercise period. 3xTg-AD mice were obtained commercially from the Jackson Laboratory (MMRRC stock number 008880) and were maintained by breeding in our facilities. Genotypes were determined by PCR analysis of DNA collected from tail biopsies. BrdU (Sigma, St. Louis, MO, USA) was administered intraperitoneally (i.p.) at 100 mg/kg/day for 7 days, and the mice were sacrificed 4 weeks after the first day of BrdU injection to observe neurogenesis.

### 4.2. Plasma Donation Mice EXERCISE Protocol

The 4-week-old mice (C57BL/6) that would eventually be used for blood donation exercised on a treadmill for animals at an inclination of 0°. For the first 4 weeks, the animals performed 5 min of warm-up exercise at a speed of 3 m/min, followed by 30 min of the main exercise at a speed of 10 m/min, and finally 5 min of a cool-down exercise at a speed of 3 m/min. Thereafter, the exercise load of the main exercise was gradually increased to a speed of 11 m/min in weeks 5–6, 12 m/min in weeks 7–8, 13 m/min in weeks 9–10, and eventually 50 min of 14 m/min in weeks 11–12. Exercise was performed five times per week, for a total of 12 weeks.

### 4.3. Infusion of Young Plasma and Young Plasma from Exercised Mice

For young plasma infusion, we performed plasma infusion immediately after sacrificing 20, 4-month-old male donor mice each from the exercise and non-exercise groups, two mice every 3 days. As described previously [15], the plasma was isolated by centrifugation and injected into the tail veins of 3xTg-AD mice in 100-µL doses, one dose every 3 days, for a total of 10 doses.

### 4.4. Behavioral Analysis

#### 4.4.1. Morris Water Maze

Spatial learning and memory were analyzed using the Morris water maze task. One day before starting training, for acclimation, the animals were made to swim freely in the swimming pool for 60 s without an escape platform. Training (learning) consisted of the animals trying to find an escape platform, and was performed four times a day for 5 days. Animals that could not find the escape platform within 60 s were guided to the platform, while swimming, by the experimenter. The animals were allowed to rest for 30 s on the platform. A probe trial was performed 24 h after the last training session, in which animals were allowed to swim freely for 60 s with no escape platform. Video tracking was used to automatically measure how well the animals remembered the previous location of the escape platform.

#### 4.4.2. Step-Through Avoidance Test

Long-term memory was measured using the step-through avoidance test. On the first day of training, the animals were placed on a platform brightly lit by a halogen lamp, and the door to a box was left open. Once the animal entered the box, the door was closed and the animal was allowed to remain inside the box for 20 s. This method was repeated twice. Finally, on the third trial, as soon as the door was closed, the animal was given a single 0.3 mA electrical shock for 2 s through the floor. After 24 h, the animal was placed on the brightly lit platform, and after the door to the box was opened, the time was measured until the animal entered the box (latency). The latency was measured up to 300 s, and animals that did not enter the box within this time were scored a latency of 300 s.

### 4.5. Preparation of Tissue

The animals were euthanized immediately after the water maze test. To prepare the brain slices, the mice were fully anesthetized with ethyl ether, perfused transcardially with 50 mM phosphate-buffered saline (PBS), and then fixed with a freshly prepared solution of 4% paraformaldehyde in 100 mM phosphate buffer (pH 7.4). The brains were then removed, post-fixed in the same fixative overnight, and transferred into a 30% sucrose solution for cryoprotection. Coronal sections with a thickness of 40 μm were created using a freezing microtome (Leica, Nussloch, Germany). From each group of 10 animals, five were used for immunohistochemistry and five for Western blot and mitochondrial function analysis. The hippocampal tissue for Western blot analysis was immediately stored at −70 °C until use. For immunohistochemistry, two sections from each group were analyzed, resulting in a total of 10 slices. Western blot was performed to analyze all five samples in each group and then was re-quantified for each protein and reanalyzed in the five samples. Therefore, the density value was calculated and analyzed a total of 10 times in each group.

### 4.6. Immunohistochemistry

To visualize cell proliferation expression, immunohistochemistry for doublecortin (DCX) in the dentate gyrus was performed. The sections were incubated in PBS for 10 min, and then washed three times for 5 min in the PBS. The sections were then incubated in 1% H_2_O_2_ for 30 min. The sections were selected from each brain and incubated overnight with goat anti-DCX antibody (1:500; Santa cruz, Dallas, TX, USA) and then with biotinylated secondary antibody (rabbit) (1:250; Vector Laboratories, Burlingame, CA, USA) for another 1.5 h. Signal from the secondary antibody was amplified with the Vector Elite ABC kit^®^ (1:100; Vector Laboratories). Antibody-biotin-avidin-peroxidase complexes were visualized using a DAB substrate kit (Vector Laboratories). The slides were air-dried overnight at room temperature, and the coverslips were mounted using Permount ^®^ (Fisher scientific, Fair lawn, NJ, USA).

### 4.7. Immunofluorescence

BrdU/NeuN-positive cells in the dentate gyrus were tested for immunofluorescence. In brief, the brain sections were permeabilized by incubation in 0.5% Triton X-100 in PBS for 20 min, then incubated in 50% formamide-2× standard saline citrate at 65 °C for 2 h, denaturated in 2 N HCl at 37 °C for 30 min, and rinsed twice in 100 mM sodium borate (pH 8.5). The sections were incubated overnight with a rat anti-BrdU antibody (1:500; Abcam, Cambridge, UK) and mouse anti-NeuN antibody (1:500; Millipore, Temecula, CA). The brain sections were then washed in PBS and incubated with the appropriate secondary antibodies for 1.5 h. The secondary antibodies used were anti-mouse IgG Alexa Fluor-488 and anti-rat IgG Alexa Fluor-550. Images were captured using an FV3000 confocal microscope (Olympus, Tokyo, Japan).

### 4.8. TUNEL Staining

To visualize DNA fragmentation, we performed TUNEL staining using an In Situ Cell Death Detection Kit (Roche Diagnostics, Risch-Rotkreuz, Switzerland) according to the manufacturer’s protocol. The sections were post-fixed in ethanol-acetic acid (2:1), rinsed, incubated with proteinase K (100 mg/mL), and then rinsed again. Next, the sections were incubated in 3% H_2_O_2_, permeabilized with 0.5% Triton X-100, rinsed again, and incubated in the TUNEL reaction mixture. The sections were rinsed and visualized using Converter-POD with 0.03% DAB, counterstained with Nissl, and mounted onto gelatin-coated slides. The slides were air-dried overnight at room temperature and cover-slipped using Permount mounting medium.

### 4.9. Western Blotting

The hippocampal tissues were homogenized on ice and lysed in a lysis buffer containing 50 mM Tris–HCl (pH 7.5), 150 mM NaCl, 0.5% deoxycholic acid, 1% Nonidet P40, 0.1% sodium dodecyl sulfate, 1 mM PMSF, and leupeptin 100 mg/mL. The protein content was measured using a colorimetric protein assay kit (Bio-Rad, Hercules, CA, USA). Thirty micrograms of protein were separated on sodium dodecyl sulfate-polyacrylamide gels and transferred onto a nitrocellulose membrane, which was incubated with antibodies against β-actin (1:2000; Santa Cruz Biotechnology), GAPDH (1:2000; Santa Cruz Biotechnology), t-GSK3β, p-GSK3β (ser 9) (1:1000; Cell Signaling), t-Tau, p-Tau (ser262) (1:1000; Thermo Fisher), p-tau (thr 205) (1:1000; Thermo Fisher), Bcl-2 (1;1000; Santa Cruz Biotechnology), cytochrome c (1:1000; Santa Cruz Biotechnology), Bax (1:1000; Cell Signaling), cleaved caspase-3,-9 (1:700; Cell Signaling), BDNF (1:1000; Alomone), PSD95 (1:1000; Cell Signaling), and synaptophysin (1:1000; Abcam). Horseradish peroxidase-conjugated anti-mouse antibodies for Bcl-2, p-Tau, cytochrome c, β-actin and GAPDH, and anti-rabbit antibodies for t-tau, t-GSK3β, p-GSK3β, Bax, cleaved caspase-3, BDNF, PSD95, and synaptophysin were used as secondary antibodies.

### 4.10. Mitochondrial Ca^2+^ Retention Capacity

The mitochondrial calcium retention capacity was tested to assess the susceptibility of the permeability transition pore (PTP) to opening. Briefly, after grinding the hippocampal tissue, overlaid traces of changes in fluorescence induced by Calcium Green-5 N were measured continuously (ΔF/min) at 37 °C during state 4 respiration using a Spex FluoroMax 4 spectrofluorometer (Horiba Scientific, Edison, NJ, USA). After establishing the background ΔF (hippocampal tissue in the presence of 1 μM Calcium Green-5 N, 1 U/mL hexokinase, 0.04 mM EGTA, 1.5 nM thapsigargin, 5 mM 2-deoxyglucose, 5 mM glutamate, 5 mM succinate, and 2 mM malate), the reaction was initiated by addition of Ca^2+^ pulses (12.5 nM), with excitation and emission wavelengths set at 506 and 532 nm, respectively. The total mitochondrial calcium retention capacity prior to PTP opening (i.e., release of Ca^2+^) was expressed as pmol/mg tissue weight.

### 4.11. Mitochondrial H_2_O_2_ Emission

The mitochondrial H_2_O_2_ emission was measured at 37 °C (ΔF/min) during state 4 respiration (10 μg/mL oligomycin) by continuously monitoring oxidation of Amplex Red (excitation/emission λ = 563/587 nm) using a Spex FluoroMax 4 spectrofluorometer with the following protocol: 10 μM Amplex Red, 1 U/mL horseradish peroxidase, and 10 μg/mL oligomycin; followed by 1 mM malate + 2 mM glutamate (complex I substrates); 3 mM succinate (complex II substrate); and 10 mM glycerol-3-phosphate (lipid substrate). The mitochondrial H_2_O_2_ emission rate after removing the background value from each of the standard values (standard curve) was calculated from the ΔF/min gradient values and expressed as pmol/min/mg tissue weight.

### 4.12. Plasma BDNF Analysis in Donation Mice

The plasma BDNF concentration was measured using ELISA. The ELISA kit was purchased from R & D Biology Inc. (Total BDNF Quantikine ELISA kit DBNT00, R & D Systems), and the experiment was performed strictly according to the manufacturer’s instructions. The specific protocol was as follows: first, the reagents, samples, and standard products were prepared. The samples and standard products were added and reacted for 120 min at 37 °C. After washing the plate three times, antibody working solution was added and the mixture was left to react for 60 min at 37 °C. The plate was washed three times again, horseradish peroxidase (HRP) was added, and the mixture was left to react for 30 min at 37 °C. After washing the plate three times, substrate working solution was added, and the mixture was left to react in a dark place for 5–10 min. After adding stop buffer, a BioTek ELx800 full-automatic enzyme labeling system ( Winooski, VT, USA) was used to detect the optical density (OD) at 450 nm within 30 min.

### 4.13. Statistical Analysis

Cell counting and optical density quantification for TUNEL, DCX, and BrdU/NeuN-positive cells were performed using Image-Pro^®^ Plus (Media Cyberbetics Inc. Rockville, MD, USA) attached to a light microscope (Olympus, Tokyo, Japan). The data were analyzed with one-way ANOVA, followed by Tukey post-hoc tests. Plasma BDNF was analyzed using *t*-tests. All values are expressed as the mean ± standard error of the mean (S.E.M.), and *p*-values < 0.05 were considered significant.

## 5. Conclusions

Treatment with plasma from young control or exercised mice showed positive effects on cognitive function in a 3xTg-AD animal model, with partial improvements in hippocampal neuroplasticity and mitochondrial function. In particular, in plasma from young exercised mice, elevated plasma BDNF levels, as a result of exercise, might have a protective effect against cognitive dysfunction and on important AD-related neural pathways, acting via tau-independent mechanisms. Further studies will be needed to investigate potential mechanisms mediating these interactions.

## Abbreviations

AD	Alzheimer’s disease
Aβ	Amyloid beta
BDNF	Brain-derived neurotrophic factor
BrdU	5-Bromo-2’-Deoxyuridine
DAB	3,3′-Diaminobenzidine
DCX	Doublecortin
EYP	Exercised young plasma
GM	Glutamate + malate
GM+S	GM + succinate
GMS+G3P	GMS + glycerol-3-phosphate
GSK-3β	Glycogen synthase kinase-3 beta
NeuN	Neuronal nuclei
PBS	phosphate-buffered saline
PSD95	postsynaptic density protein 95
ROS	Reactive oxygen species
TUNEL	Terminal deoxynucleotidyl transferase dUTP nick end labeling
YP	Young plasma
3xTg-AD	Triple-transgenic mouse model-AD

## References

[1] Mattson M.P. (2004). Pathways towards and away from Alzheimer’s disease. Nature.

[2] Swerdlow R.H., Khan S.M. (2009). The Alzheimer’s disease mitochondrial cascade hypothesis: An update. Exp. Neurol..

[3] Kumar A., Singh A., Ekavali (2015). A review on Alzheimer’s disease pathophysiology and its management: An update. Pharmacol. Rep..

[4] Blennow K., de Leon M.J., Zetterberg H. (2006). Alzheimer’s disease. Lancet.

[5] Arendt T. (2009). Synaptic degeneration in Alzheimer’s disease. Acta Neuropathol..

[6] Siwak-Tapp C.T., Head E., Muggenburg B.A., Milgram N.W., Cotman C.W. (2007). Neurogenesis decreases with age in the canine hippocampus and correlates with cognitive function. Neurobiol. Learn. Mem..

[7] Stamer K., Vogel R., Thies E., Mandelkow E., Mandelkow E.M. (2002). Taublockstraffic of organelles, neurofilaments, and APPvesicles in neurons and enhance soxidativestress. Cell Biol..

[8] Mandelkow E.M., Stamer K., Vogel R., Thies E., Mandelkow E. (2003). Clogging of axons by tau, inhibition of axonaltraffic and starvation of synapses. Neurobiol. Aging.

[9] Dubey M., Chaudhury P., Kabiru H., Shea T.B. (2008). Tau inhibits anterograde axonal transport and perturbs stability in growing axonal neurites in part by displacing kinesin cargo: Neurofilaments attenuate tau-mediated neurite instability. Cell Motil. Cytoskelet..

[10] Bordone M.P., Salman M.M., Titus H.E., Amini E., Andersen J.V., Chakraborti B., Diuba A.V., Dubouskaya T.G., Ehrke E., Espindola de Freitas A. (2019). The energetic brain—A review from students to students. J. Neurochem..

[11] Gibson G.E., Sheu K.F., Blass J.P. (1998). Abnormalities of mitochondrial enzymes in Alzheimer disease. J. Neural Transm. (Vienna).

[12] Devi L., Prabhu B.M., Galati D.F., Avadhani N.G., Anandatheerthavarada H.K. (2006). Accumulation of amyloid precursor protein in the mitochondrial import channels of human Alzheimer’s disease brain is associated with mitochondrial dysfunction. J. Neurosci..

[13] Parker W.D., Filley C.M., Parks J.K. (1990). Cytochrome oxidase deficiency in Alzheimer’s disease. Neurology.

[14] Wang X., Su B., Siedlak S.L., Moreira P.I., Fujioka H., Wang Y., Casadesus G., Zhu X. (2008). Amyloid-beta overproduction causes abnormal mitochondrial dynamics via differential modulation of mitochondrial fission/fusion proteins. Proc. Natl. Acad. Sci. USA.

[15] Villeda S.A., Plambeck K.E., Middeldorp J., Castellano J.M., Mosher K.I., Luo J., Smith L.K., Bieri G., Lin K., Berdnik D. (2014). Young blood reverses age-related impairments in cognitive function and synaptic plasticity in mice. Nat. Med..

[16] Kim D.H., Ko I.G., Kim B.K., Kim T.W., Kim S.E., Shin M.S., Kim C.J., Kim H., Kim K.M., Baek S.S. (2010). Treadmill exercise inhibits traumatic brain injury-induced hippocampal apoptosis. Physiol. Behav..

[17] Luo C.X., Jiang J., Zhou Q.G., Zhu X.J., Wang W., Zhang Z.J., Han X., Zhu D.Y. (2007). Voluntary exercise-induced neurogenesis in the postischemic dentate gyrus is associated with spatial memory recovery from stroke. J. Neurosci. Res..

[18] Ke Z., Yip S.P., Li L., Zheng X.X., Tong K.Y. (2011). The effects of voluntary, involuntary, and forced exercises on brain-derived neurotrophic factor and motor function recovery: A rat brain ischemia model. PLoS ONE.

[19] Lee S.U., Kim D.Y., Park S.H., Choi D.H., Park H.W., Han T.R. (2009). Mild to moderate early exercise promotes recovery from cerebral ischemia in rats. Can. J. Neurol. Sci..

[20] Zoladz J.A., Pilc A., Majerczak J., Grandys M., Zapart-Bukowska J., Duda K. (2008). Endurance training increases plasma brain-derived neurotrophic factor concentration in young healthy men. J. Physiol. Pharmacol..

[21] Seifert T., Brassard P., Wissenberg M., Rasmussen P., Nordby P., Stallknecht B., Adser H., Jakobsen A.H., Piegaard H., Nielsen H.B. (2010). Endurance training enhances BDNF release from the human brain. Am. J. Physiol.-Regul. Integr. Comp. Physiol..

[22] Salman M.M., Kitchen P., Woodroofe M.N., Bill R.M., Conner A.C., Heath P.R., Conner M.T. (2017). Transcriptome Analysis of Gene Expression Provides New Insights into the Effect of Mild Therapeutic Hypothermia on Primary Human Cortical Astrocytes Cultured under Hypoxia. Front. Cell. Neurosci..

[23] Chun H., Marriott I., Lee C.J., Cho H. (2018). Elucidating the Interactive Roles of Glia in Alzheimer’s Disease Using Established and Newly Developed Experimental Models. Front. Neurol..

[24] Heneka M.T., Carson M.J., El Khoury J., Landreth G.E., Brosseron F., Feinstein D.L., Jacobs A.H., Wyss-Coray T., Vitorica J., Ransohoff R.M. (2015). Neuroinflammation in Alzheimer’s disease. Lancet Neurol..

[25] Oddo S., Caccamo A., Shepherd J.D., Murphy M.P., Golde T.E., Kayed R., Metherate R., Mattson M.P., Akbari Y., LaFerla F.M. (2003). Triple-transgenic model of Alzheimer’s disease with plaques and tangles: Intracellular Abeta and synaptic dysfunction. Neuron.

[26] Carroll J.C., Rosario E.R., Chang L., Stanczyk F.Z., Oddo S., LaFerla F.M., Pike C.J. (2007). Progesterone and estrogen regulate Alzheimer-like neuropathology in female 3xTg-AD mice. J. Neurosci..

[27] Reddy P.H. (2011). Abnormal tau, mitochondrial dysfunction, impaired axonal transport of mitochondria, and synaptic deprivation in Alzheimer’s disease. Brain Res..

[28] Atlante A., Amadoro G., Bobba A., de Bari L., Corsetti V., Pappalardo G., Marra E., Calissano P., Passarella S. (2008). A peptide containing residues 26–44 of tau protein impairs mitochondrial oxidative phosphorylation acting at the level of the adenine nucleotide translocator. Biochim. Biophys. Acta.

[29] Kristian T., Pivovarova N.B., Fiskum G., Andrews S.B. (2007). Calcium-induced precipitate formation in brain mitochondria: Composition, calcium capacity, and retention. J. Neurochem..

[30] Tsujimoto Y., Shimizu S. (2007). Role of the mitochondrial membrane permeability transition in cell death. Apoptosis.

[31] Villeda S.A., Luo J., Mosher K.I., Zou B., Britschgi M., Bieri G., Stan T.M., Fainberg N., Ding Z., Eggel A. (2011). The ageing systemic milieu negatively regulates neurogenesis and cognitive function. Nature.

[32] Yuan J.J., Zhang Q., Gong C.X., Wang F.X., Huang J.C., Yang G.Q., Liu L., Zhou K., Xu R., Chen Q. (2019). Young plasma ameliorates aging-related acute brain injury after intracerebral hemorrhage. Biosci. Rep..

[33] Middeldorp J., Lehallier B., Villeda S.A., Miedema S.S., Evans E., Czirr E., Zhang H., Luo J., Stan T., Mosher K.I. (2016). Preclinical Assessment of Young Blood Plasma for Alzheimer Disease. JAMA Neurol..

[34] Belviranlı M., Okudan N. (2018). Exercise training increases cardiac, hepatic and circulating levels of brain-derived neurotrophic factor and irisin in young and aged rats. Horm. Mol. Biol. Clin. Investig..

[35] Gilder M., Ramsbottom R., Currie J., Sheridan B., Nevill A.M. (2014). Effect of fat free mass on serum and plasma BDNF concentrations during exercise and recovery in healthy young men. Neurosci. Lett..

[36] Qin X.Y., Cao C., Cawley N.X., Liu T.T., Yuan J., Loh Y.P., Cheng Y. (2017). Decreased peripheral brain-derived neurotrophic factor levels in Alzheimer’s disease: A meta-analysis study (N = 7277). Mol. Psychiatry.

[37] Nagata T., Kobayashi N., Shinagawa S., Yamada H., Kondo K., Nakayama K. (2014). Plasma BDNF levels are correlated with aggressiveness in patients with amnestic mild cognitive impairment or Alzheimer disease. J. Neural. Transm. (Vienna).

[38] Klein A.B., Williamson R., Santini M.A., Clemmensen C., Ettrup A., Rios M., Knudsen G.M., Aznar S. (2011). Blood BDNF concentrations reflect brain-tissue BDNF levels across species. Int. J. Neuropsychopharmacol..

[39] Nagahara A.H., Merrill D.A., Coppola G., Tsukada S., Schroeder B.E., Shaked G.M., Wang L., Blesch A., Kim A., Conner J.M. (2009). Neuroprotective effects of brain-derived neurotrophic factor in rodent and primate models of Alzheimer’s disease. Nat. Med..

[40] Jiao S.S., Shen L.L., Zhu C., Bu X.L., Liu Y.H., Liu C.H., Yao X.Q., Zhang L.L., Zhou,  H.D., Walker D.G. (2016). Brain-derived neurotrophic factor protects against tau-related neurodegeneration of Alzheimer’s disease. Transl. Psychiatry.

[41] Sairanen M., Lucas G., Ernfors P., Castrén M., Castrén E. (2005). Brain-derived neurotrophic factor and antidepressant drugs have different but coordinated effects on neuronal turnover, proliferation, and survival in the adult dentate gyrus. J. Neurosci..

[42] Han B.H., D’Costa A., Back S.A., Parsadanian M., Patel S., Shah A.R., Giddy J.M., Srinivasan A., Deshmukh M., Holtzman D.M. (2000). BDNF blocks caspase-3 activation in neonatal hypoxia-ischemia. Neurobiol. Dis..

[43] Markham A., Cameron I., Franklin P., Spedding M. (2004). BDNF increases rat brain mitochondrial respiratory coupling at complex I, but not complex II. Eur. J. Neurosci..

